# Morphine-3-Glucuronide, Physiology and Behavior

**DOI:** 10.3389/fnmol.2022.882443

**Published:** 2022-05-12

**Authors:** Florian Gabel, Volodya Hovhannisyan, Abdel-Karim Berkati, Yannick Goumon

**Affiliations:** ^1^CNRS UPR 3212, Institut des Neurosciences Cellulaires et Intégratives, Centre National de la Recherche Scientifique and University of Strasbourg, Strasbourg, France; ^2^SMPMS, Mass Spectrometry Facilities of the CNRS UPR 3212, Institut des Neurosciences Cellulaires et Intégratives, Centre National de la Recherche Scientifique, Strasbourg, France

**Keywords:** morphine, metabolism, M3G, hyperalgesia, MOR – mu opioid receptor, TLR4 – toll-like receptor 4

## Abstract

Morphine remains the gold standard painkiller available to date to relieve severe pain. Morphine metabolism leads to the production of two predominant metabolites, morphine-3-glucuronide (M3G) and morphine-6-glucuronide (M6G). This metabolism involves uridine 5′-diphospho-glucuronosyltransferases (UGTs), which catalyze the addition of a glucuronide moiety onto the C3 or C6 position of morphine. Interestingly, M3G and M6G have been shown to be biologically active. On the one hand, M6G produces potent analgesia in rodents and humans. On the other hand, M3G provokes a state of strong excitation in rodents, characterized by thermal hyperalgesia and tactile allodynia. Its coadministration with morphine or M6G also reduces the resulting analgesia. Although these behavioral effects show quite consistency in rodents, M3G effects are much more debated in humans and the identity of the receptor(s) on which M3G acts remains unclear. Indeed, M3G has little affinity for mu opioid receptor (MOR) (on which morphine binds) and its effects are retained in the presence of naloxone or naltrexone, two non-selective MOR antagonists. Paradoxically, MOR seems to be essential to M3G effects. In contrast, several studies proposed that TLR4 could mediate M3G effects since this receptor also appears to be essential to M3G-induced hyperalgesia. This review summarizes M3G’s behavioral effects and potential targets in the central nervous system, as well as the mechanisms by which it might oppose analgesia.

## Introduction

Over the last decade, chronic pain has become one of the top health burdens threatening economic and healthcare systems (GBD 2016 Disease and Injury Incidence and Prevalence Collaborators, 2017). Opiates, such as morphine and its derivatives, remain the most potent painkillers available at the hospital. However, their use and efficiency are limited by mild (i.e., nausea, constipation) to severe side effects, including analgesic tolerance, opioid use disorders and ultimately respiratory depression, which can lead to death (Trescot et al., 2008; Koller et al., 2019; Bachmutsky et al., 2020). Among side effects, analgesic tolerance corresponds to the decreased effect of opioid-induced analgesia following repeated administrations (Trescot et al., 2008; Weinsanto et al., 2018; Gabel et al., 2022). Consequently, dose escalation is required to relieve pain, although it might result in greater risks of severe side effects. In addition, opiate efficiency and side effects are influenced by numerous factors, including sex, age (van Crugten et al., 1997; Fullerton et al., 2021; Gabel et al., 2022), comorbidities (Gupta et al., 2018), additional drug treatments and pain types (Vellucci, 2012; Hopkins et al., 2019), resulting in complex patient care (Turk, 1996; Vellucci, 2012). In particular, morphine has been extensively used to decipher the mechanisms involved in opiate-induced analgesia, tolerance and opioid use disorders.

Morphine’s effects are mediated mainly through the activation of mu opioid receptors (MORs) located in cerebral structures involved in the descending controls of pain, including the periaqueductal gray matter (PAG), the rostral ventromedial medulla (RVM) and the spinal cord. Upon activation, these receptors induce the hyperpolarization of MOR-expressing neurons, resulting in the inhibition of nociceptive signal transmission (for review, see Lau and Vaughan, 2014). From a pharmacokinetic point of view, after administration, morphine undergoes sequential pharmacological processes, consisting of absorption, distribution, metabolism, and excretion (ADME). Following intestinal absorption, morphine reaches the liver and enters the hepatocytes, wherein a major part of its metabolism occurs. Hence, morphine bioavailability is relatively low in humans (Hasselstrom and Sawe, 1993; Lotsch et al., 1999; Lloret-Linares et al., 2016), with only 25–35% of morphine reaching the circulation and even less being distributed within the central nervous system (CNS). Indeed, the blood-brain barrier (BBB) restrains CNS access to xenobiotics and, to a more general extent, hydrophilic compounds. The BBB is a selectively semipermeable barrier composed of adjacent endothelial cells, astrocyte end feet, and pericytes (Ballabh et al., 2004). Although morphine crosses endothelial cell membranes due to a certain degree of lipophilicity, its BBB permeability relies on the *P*-glycoprotein (*P*-gp) drug transporter, which drives morphine from endothelial cells back into the blood (Schaefer et al., 2017). Therefore, the effectiveness and duration of the analgesic effect of morphine are partially modulated by both morphine metabolism and the permeability of the BBB. Pharmacodynamic processes are also key elements affecting morphine’s effects. However, they are beyond the scope of the present review and have already been discussed elsewhere (for review, see Al-Hasani and Bruchas, 2011; Williams et al., 2013).

## Morphine Metabolism

### Glucuronidation of Morphine

Morphine metabolism involves mainly hepatic glucuronidation by uridine 5′-diphospho-glucuronosyltransferase (UGT) phase II enzymes. Glucuronidation occurs at the C3-OH and C6-OH positions, leading to two active metabolites: morphine-3-glucuronide (M3G) and morphine-6-glucuronide (M6G) (Lotsch, 2005). However, to a much lesser extent, other morphine metabolites (5%) can be found in the blood and urine and include normorphine or morphine sulfates (Yeh et al., 1977; Hand et al., 1987a; Cone et al., 2008; Laux-Biehlmann et al., 2013). In addition, 10% of morphine is excreted in its intact form due to its intrinsic hydrophilicity (Yeh, 1975). Pharmacokinetic studies of morphine in humans have shown blood terminal half-life average values of 2–3 h in healthy patients (Hasselstrom and Sawe, 1993). However, significant variations, ranging from less than 1 h up to 7 h (Webster et al., 1976; Sawe, 1986), have been reported based on the route of administration (e.g., more prolonged for oral *vs.* intravenous) and individual physiology (e.g., age, gender, comorbidities, cotreatments).

### UDP-Glucuronosyltransferases

**FIGURE 1:**
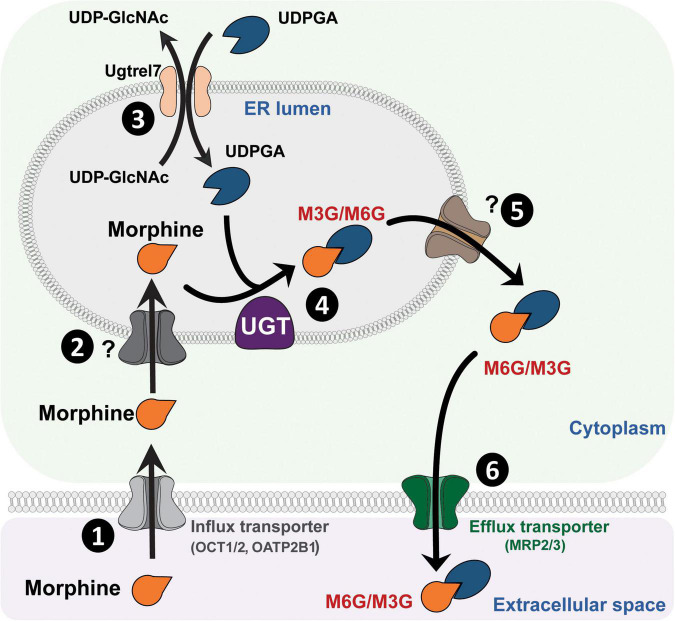
Glucuronidation process of morphine. **(1)** Morphine is first transferred from the extracellular space into the cytoplasm through active transporters such as OCT1 or OATP2B1. **(2)** Then, morphine enters the lumen of the ER by way of an unknown transporter. **(3)** Concomitantly, UDPGA is transported across the ER membrane in exchange for UDP-GlcNac, due to UGTrel transporters, such as UGTrel7. **(4)** Inside the lumen, UGT catalyzes the addition of a glucuronide moiety belonging to UDPGA onto morphine to form M3G and M6G. **(5)** Metabolites are then transferred into the cytoplasm by unknown transporters. **(6)** Finally, they are released into the extracellular space through active transporters such as MRP2 or MRP3. It is worth noting that a significant proportion of morphine that enters the cytoplasm can be directly released into the extracellular space via P-gp. M3G, morphine-3-glucuronide; M6G, morphine-6-glucuronide; MRP, multidrug resistance-associated protein; OATP2B1, organic anion transporter polypeptides 2B1; OCT1, organic cation transporter 1; P-gp, P-glycoprotein; ER, endoplasmic reticulum; UDPGA, uridine diphosphate glucuronic acid; UDP-GlcNac, UDP-*N*-acetylglucosamine; UGT, UDP-glucuronosyltransferase.

UGTs are transmembrane glycoproteins located in the smooth endoplasmic reticulum (ER; Figure 1). These proteins are composed of approximately 550 amino acids (around 49 kDa) and represent a superfamily of enzymes divided into two groups: UGT1A and UGT2B (for review, see Meech et al., 2019). Studies using human liver microsomes have established that several UGTs are involved in morphine glucuronidation, including UGT1A1, UGT1A3, UGT1A6, UGT1A8, UGT2B1, and UGT2B7 (Stone et al., 2003; Nair et al., 2015). Among them, the UGT2B7 is considered as the main enzyme involved in morphine metabolism. These enzymes catalyze the conjugation of a nucleophilic aglycone moiety (acceptor substrate; i.e., morphine) to the glycosyl group of a nucleotide sugar (donor; i.e., uridine diphosphate glucuronic acid, UDPGA). The main transporters involved in morphine transport across cell membranes are organic cation transporter member 1 (OCT1; Figure 1; Tzvetkov et al., 2013; Meyer et al., 2019), OCT2 (Imaoka et al., 2021) and the organic anion transporter polypeptide 2B1 (OATP2B1) (Yang Z. Z. et al., 2016). UGTs are found in the ER lumen, and only 20 amino acids remain in the cytosolic side, with a di-lysine (KK) motif being responsible for their membrane retention (Jackson et al., 1990). The luminal amino-terminal part of the protein carries the substrate-binding domain, whereas the carboxy-terminal part binds the cosubstrate UDP-glucuronic acid (UDPGA). It requires that both morphine and UDPGA be transported inside the ER. While the transport of UDPGA relies on several ER transporters, such as UGTrel7 (UDP-galactose transporter-related protein 7) (Muraoka et al., 2001; Kobayashi et al., 2006; Rowland et al., 2015) or UGTrel1 (Ondo et al., 2020), there is currently no identified transporter for aglycones and conjugated compounds in general or for morphine in particular. Once morphine glucuronides are transported back into the cytosol, plasma membrane efflux multidrug resistance-associated protein 2 and 3 transporters (MRP2 and MRP3) (Zelcer et al., 2005; Lloret-Linares et al., 2016), located on the basolateral side of hepatocytes, allow for their release outside the cell. Then, M3G and M6G are likely to be taken up and released into the bloodstream by endothelial cells via a probenecid-sensitive transport system (Xie et al., 2000). From the bloodstream, they reach the kidneys to be excreted in urine.

UGTs have been widely conserved across evolution from bacteria to plants and mammals (King et al., 2000; Bock, 2016). However, notable differences exist in enzyme expression and morphine metabolism between species (Oda et al., 2015). For instance, major disparities in morphine metabolism have been reported between humans and rodents. In particular, morphine has a shorter terminal half-life in C57BL/6 mice than in humans (i.e., 30 min *vs.* few hours in humans) (Handal et al., 2002). In humans, M3G and M6G represent 90 and 10% of all glucuronidated metabolites, respectively (Hasselstrom and Sawe, 1993). Alternatively, mice exclusively convert morphine into M3G due to the lack of UGT2B7 expression. Indeed, UGT2B7 seems to be required for M6G production, as witnessed in several *in vitro* studies using human and rodent microsomes (Lotsch and Geisslinger, 2001; Stone et al., 2003; for review, see Court, 2005). One hypothesis could be that the piperidine ring bearing the tertiary amine N17 disturbs the interaction between other UGTs and the C6-OH position of morphine. Thus, it might significantly decrease the glucuronidation probability at this position. Nevertheless, a baculovirus-Sf9 cell system for expressing UGTs, established by Kurita et al. (2017), demonstrated that UGT2B36 is the main M3G-forming enzyme in male FVB mice. In addition, although UGT1A1 and UGT2B1 individually did not form M3G in one particular study, heterodimers of these isoforms produced high levels of M3G (Miyauchi et al., 2020). In summary, although a few reports have suggested that rodents which lack UGT2B7 (Shelby et al., 2003; Buckley and Klaassen, 2007) might produce low levels of M6G (Nagano et al., 2000; Togna et al., 2013; Yang et al., 2016a,b; Yang Z. Z. et al., 2016), the general consensus is that they cannot synthesize such metabolites (Oguri et al., 1990; Kuo et al., 1991; Salem and Hope, 1997; Zelcer et al., 2005; Dahan and Lotsch, 2015; Allette et al., 2017; Gabel et al., 2022).

Since the beginning of the 1970s, the predominant metabolites of morphine, M3G and M6G, have been shown to be biologically active. On the one hand, M6G binds to MOR with high affinity and produces potent analgesia. On the other hand, M3G has been described as having pronociceptive properties that could counteract morphine and M6G analgesia. This review focuses on what is known about M3G behavioral effects, its potential targets in the CNS and the mechanisms underlying its properties. M6G effects are beyond the scope of this review and have already been discussed elsewhere (for review, see Lotsch and Geisslinger, 2001).

## Morphine-3-Glucuronide

### Behavioral Observations

#### Rodents

The predominant morphine metabolite, M3G, is devoid of analgesic effects whether it is injected through the s.c. or i.c.v. routes or directly into the PAG (Shimomura et al., 1971; Pasternak et al., 1987). However, the first M3G administrations in rats have elicited neuroexcitatory effects that could oppose morphine and M6G antinociception (Table 1). For instance, i.t. or i.c.v. administration of M3G induces robust behavioral excitation in rodents characterized by spontaneous agitation, hyperalgesia and allodynia, epileptic episodes and death following high doses of M3G (Labella et al., 1979; Woolf, 1981; Yaksh et al., 1986; Yaksh and Harty, 1988; Bartlett et al., 1994a; Bian and Bhargava, 1996). Following these observations, M3G’s pronociceptive effects were evaluated after direct injection or when coadministered with morphine or M6G. In rodents, i.p., s.c. and i.t. injections of M3G alone clearly induce thermal hyperalgesia and mechanical allodynia (Juni et al., 2006; Komatsu et al., 2009, 2016; Lewis et al., 2010; Due et al., 2012; Arout et al., 2014; Allette et al., 2017; Roeckel et al., 2017; Blomqvist et al., 2020). Additionally, morphine and M6G analgesic effects are markedly reduced by M3G (Smith et al., 1990; Qian-Ling et al., 1992; Ekblom et al., 1993; Faura et al., 1996, 1997; Gardmark et al., 1998). Hence, whether it is injected alone or with morphine or M6G, several studies have demonstrated that M3G has pronociceptive properties in rodents (Table 1).

**TABLE 1:** M3G behavioral studies in *favor* of M3G pronociceptive effects.

References	Agonist	Administration type	Species	M3G effects
Labella et al., 1979	M3G	i.c.v infusion	SD male rats	Behavioral excitation
Yaksh et al., 1986	M3G	i.t. (3 μg)	Rats and cats	
Bartlett et al., 1994a	M3G	i.c.v. (2–8 μg)	SD male rats	
Bian and Bhargava, 1996	M3G	i.c.v. (3 and 10 μg)	SW male mice	
Komatsu et al., 2009	M3G	i.t. (3 nmol)	ddY male mice	
Komatsu et al., 2016	M3G	i.t. (2.5 nmol)	ddY male mice	

Woolf, 1981	M3G	i.t. (15 μg)	SD male rats	Thermal hyperalgesia and/or mechanical allodynia
Smith et al., 1990	M3G	i.c.v. (2.5 and 3 μg)/i.p. (10 mg/kg)	SD male rats	
Gong et al., 1991	M3G	i.c.v. (0.2 to 8–10 μg)	SD male rats	
Juni et al., 2006	M3G	s.c. infusion (5 mg/kg)	CD-1 male mice	
Lewis et al., 2010	M3G	i.t. (0.75 μg)	SD male Rats	
Due et al., 2012	M3G	i.p. (25 mg/kg)	SD female rats and C57BL/10ScNJ TLR4^–/–^ male mice	
Arout et al., 2014	M3G	s.c. (5 mg/kg)	CD-1 male mice	
Allette et al., 2017	M3G	i.p. (concentration not stated)	C57BL/6J female mice	
Roeckel et al., 2017	M3G	i.p. (5 mg/kg)	Male and female 50% C57/BL6J:50% 129svPas mice	
Blomqvist et al., 2020	M3G	i.t. (5 μg)	SD male rats	

Smith et al., 1990	M3G + M/M3G + M6G	i.c.v. (2.5 and 3 μg)/i.p. (10 mg/kg)	SD male rats	M3G-mediated decrease of morphine and/or M6G analgesia
Qian-Ling et al., 1992	M3G + M6G	i.c.v. (0.5 μg) i.t. (0.5 μg)	SD male rats	
Ekblom et al., 1993	M3G + M	i.v. (9.4 μmol/h/kg M3G, 35 μmol/h/kg M)	SD male rats	
Faura et al., 1996	M3G + M6G	s.c. (6 mg/kg M3G, 4 mg/kg M6G)	SW male mice	
Faura et al., 1997	M3G + M6G	s.c. (6 mg/kg M3G, 4 mg/kg M6G)	SW male mice	
Gardmark et al., 1998	M3G + M	M3G infusion overnight (9.4 or 37.6 μmol/h/kg) then morphine infusion	SD male rats	
Mazoit et al., 2007	M	i.v. (bolus 10 mg)	50 patients with pain	
Doyle and Murphy, 2018	M3G + M	Intra-vlPAG injection (0.075 μg/0.25μl/side) followed by s.c. M	SD male and female rats	

Smith and Smith, 1995	M	Continuous i.v. infusion (3 different dosing regimes)	SD male rats	M3G/Morphine ratio inversely correlated to morphine antinociception
Barjavel et al., 1995	M	s.c. (10 mg/kg)	SD male rats	
Smith et al., 1999	M	Oral or s.c. then i.c.v. postventriculostomy	14 patients	

Morley et al., 1992	M	i.t. + oral M then M6G (1 mg) i.t.	One 47 years old man	High levels of M3G in CSF corroborated with worsened pain

Sjogren et al., 1993	M	Continuous i.v. infusion (100 mg/h)	One 50 years old man	Myoclonic spasms

*The indicated concentrations for studies in which several agonists were used correspond to M3G concentrations, unless otherwise stated. CSF, cerebrospinal fluid; ddY, Deutschland, Denken, and Yoken mice; FVB, friend leukemia virus B mice; M, morphine; M3G, morphine-3-glucuronide; M6G, morphine-6-glucuronide; MRP, multidrug resistance associated protein; PK-PD, pharmacokinetic-pharmacodynamic; SD, Sprague-Dawley; SW, Swiss-Webster; TLR4, Toll-like receptor 4; vl-PAG, ventrolateral periaqueductal gray.*

Interestingly, Smith and Smith (1995) observed that, when morphine is infused continuously in rats, the higher the plasmatic metabolic ratio M3G/morphine is, the lower the antinociception is, independently of the M3G or morphine plasmatic concentrations. Similar observations were made in the extracellular cortical fluid following s.c. administration of morphine (Barjavel et al., 1995). Consequently, M3G was proposed to counteract morphine-induced analgesia and to produce neuroexcitatory effects responsible for some morphine side effects (Gong et al., 1991; Smith and Smith, 1995; Faura et al., 1997; Roeckel et al., 2017; Blomqvist et al., 2020).

Although a considerable number of studies have indicated that M3G possesses pronociceptive properties, some studies did not observe pronociceptive effects following M3G administration or when it was coadministered with morphine or M6G (Table 2). For instance, Ouellet and Pollack (1997) observed that, when M3G was infused for 12 h in rats, there was no hyperalgesia or modulation of morphine analgesia (Suzuki et al., 1993). In another study, it was even noted that the i.v. coadministration of morphine and M3G improved morphine analgesia (Lipkowski et al., 1994).

**TABLE 2:** M3G behavioral studies in opposition to M3G pronociceptive effects.

References	Agonist	Administration type	Species	M3G effects
Ekblom et al., 1993	M3G	i.v. bolus (86.7 μmol/kg)	SD male rats	No behavioral effect/hyperalgesia observed following M3G administration alone
Bian and Bhargava, 1996	M3G	i.p. (10–100 mg/kg) i.c.v. (0–2 μg)	SW male mice	
Faura et al., 1996	M3G	s.c. (6 mg/kg)	SW male mice	
Faura et al., 1997	M3G	s.c. (6 mg/kg)	SW male mice	
Salem and Hope, 1997	M3G	i.p. (2.5, 5, and 10 mg/kg)	Winstar female rats	
Ouellet and Pollack, 1997	M3G	M3G infusion (0.15 or 0.30 mg/hr)	SD male rats	
Penson et al., 2000	M3G	i.v. (30.6 mg/70 kg)	10 healthy volunteers	
Penson et al., 2001	M3G	i.v. (7.5, 15, and 30 mg/70 kg)	3 healthy volunteers/dose	

Suzuki et al., 1993	M3G + M/M3G + M6G	i.t. (5 μg)	Wistar male rats	No modulation of morphine or M6G antinociception/side effects by M3G
Bian and Bhargava, 1996	M3G + M	i.p. (10–100 mg/kg) i.c.v. (0-2 μg)	SW male mice	
Ouellet and Pollack, 1997	M3G + M	M3G infusion (0.15 or 0.30 mg/hr) then i.v. M 2 mg/kg	SD male rats	
Penson et al., 2000	M3G + M/M3G + M6G	i.v. (30.6 mg/70 kg)	10 healthy volunteers	
Zelcer et al., 2005	M	i.p. (15 mg/kg)	FVB MRP3^–/–^ mice	
Swartjes et al., 2012	M + naltrexone	s.c. (15 mg/kg each)	FVB MRP3 ^–/–^ mice	

Samuelsson et al., 1993	M	Epidural	35 cancer patients	No correlation between analgesia and the plasma or CSF M3G/(morphine or M6G) ratio
Goucke et al., 1994	M	Oral or s.c.	11 cancer patients	
Wolff et al., 1995	M	Chronic oral (slow-release)	34 cancer patients	
Wolff et al., 1996	M	Chronic s.c.	21 cancer patients	
Andersen et al., 2002	M	Chronic oral	1 cancer patient	

Lipkowski et al., 1994	M3G + M	i.v. (M3G: 10 μmol/kg and M: 2.6 μmol/kg)	SD male rats	Improved analgesia and attenuation of antinociceptive tolerance

Toce et al., 2019	M	i.v. (2 mg)	One 12 years-old boy with acute pain	Low morphine metabolism associated with an increase of morphine side effects

*The indicated concentrations for studies in which several agonists were used correspond to M3G concentrations, unless otherwise stated. CSF, cerebrospinal fluid; ddY, Deutschland, Denken, and Yoken mice; FVB, friend leukemia virus B mice; M, morphine; M3G, morphine-3-glucuronide; M6G, morphine-6-glucuronide; MRP, multidrug resistance associated protein; PK-PD, pharmacokinetic-pharmacodynamic; SD, Sprague-Dawley; SW, Swiss-Webster; TLR4, Toll-like receptor 4; vl-PAG, ventrolateral periaqueductal gray.*

Interestingly, in a MRP3^–/–^ mouse model, the antinociception and hyperalgesia induced by an injection of morphine remained intact (Swartjes et al., 2012). In these mice, although morphine is still metabolized into M3G, M3G has been shown to remain trapped in hepatocytes due to the lack of the MRP3 efflux transporter. Therefore, plasma levels of M3G were extremely low in these transgenic animals compared to control animals (Zelcer et al., 2005; Swartjes et al., 2012). These data indicate that hyperalgesia may occur without significant contribution of hepatic M3G. However, it is worth noting that, although M3G is not found in the blood of these animals, morphine might be directly metabolized into M3G within the CNS and could still elicit its central effects (Gabel et al., 2022).

#### Humans

In humans, there have been few reports of the pronociceptive effects of M3G (Table 1). Smith and collaborators observed in 14 cancer patients improved pain relief, which was corroborated by a decrease in the M3G/(morphine + M6G) ratios. These results indirectly suggest a pronociceptive role of M3G by reducing morphine analgesia (Smith et al., 1999). In a pharmacokinetic-pharmacodynamic study involving 50 patients with moderate to severe pain, M3G effects seemed to oppose morphine analgesia (Mazoit et al., 2007). Several case reports have also suggested that M3G might play a role in morphine’s side effects such as morphine-induced hyperalgesia and seizures following high dose of morphine. However, these observations have shown considerable heterogeneity and do not demonstrate a pronociceptive role of M3G in humans (Morley et al., 1992; Sjogren et al., 1993, 1998; Rozan et al., 1995; Hagen and Swanson, 1997; Kronenberg et al., 1998).

Contrastingly, several reports have not observed any correlation between analgesia and plasmatic concentrations of M3G or the metabolic M3G/(morphine + M6G) ratios (Table 2; Samuelsson et al., 1993; Goucke et al., 1994; Wolff et al., 1995; Andersen et al., 2002; Toce et al., 2019). In addition, there have been two studies, published by the same group, in which healthy volunteers were administered M3G to evaluate its effects in humans (Penson et al., 2000, 2001). The first study was a randomized, double-blind, placebo-controlled trial in which M3G was infused in 10 healthy volunteers. Analgesia was assessed with numerical and visual analog scales in a submaximal ischemic pain model. No M3G-induced hyperalgesia or dysphoria was observed. In addition, the coadministration of M3G along with morphine or M6G did not affect analgesia (Penson et al., 2000). In the second study, which was blinded, but not controlled, three concentrations of M3G were used, but no effect was observed (Penson et al., 2001). These two studies are extremely valuable, although the number of subjects used was relatively small for obvious reasons.

#### Potential Origin of the Behavioral Effect

Considered together, the few studies in humans are matter of debate, whereas, in rodents, reports have shown much more consistency toward the pronociceptive effects of M3G, even though these effects are not always observed. The origin of the behavioral effect of M3G might rely on its glucuronide moiety. Indeed, M3G is not the only “3-glucuronide” metabolite displaying pronociceptive effects. Several studies published by Lewis et al. (2013, 2015) showed that estradiol-3-glucuronide, as well as ethyl-glucuronide, produces hyperalgesia after i.t. administration. Interestingly, glucuronic acid injected alone also triggered a similar effect, demonstrating the importance of the glucuronide moiety in the pronociceptive effects of these molecules (Lewis et al., 2013).

Supporting this idea, other 3-glucuronide metabolites of morphine-derived compounds, such as normorphine, noroxymorphone and hydromorphone, display pronociceptive properties (Yaksh and Harty, 1988; Smith et al., 1997; Wright et al., 2001). Consistently, Peckham and Traynor (2006) showed robust sex differences in analgesia only with morphine derivatives that are conjugated into a 3-glucuronide metabolite. Importantly, these observations were not related to binding affinity, ability to activate the MOR or lipophilicity. We also recently observed that sex differences in morphine analgesia could have their origin in morphine metabolism. Indeed, morphine metabolism is higher in the female brain, resulting in higher levels of M3G in pain-related brain regions (Gabel et al., 2022).

### Pharmacological Targets

#### Mu Opioid Receptor

**FIGURE 2:**
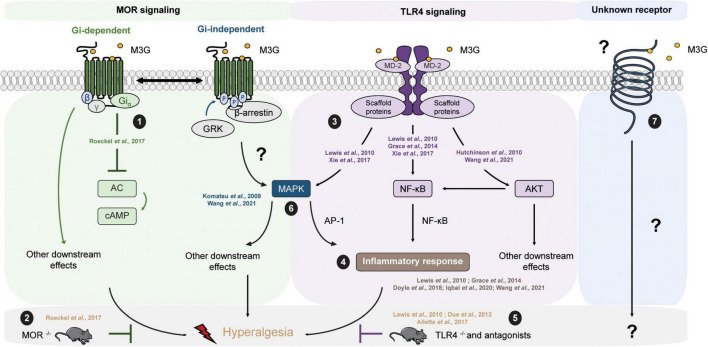
M3G known and possible intracellular pathways resulting in hyperalgesia. M3G administration causes hyperalgesia in rodents. **(1)** M3G has low affinity for MOR and has been shown to induce a weak MOR Gi-dependent signaling, although it does not seem to stimulate β-arrestin recruitment. **(2)** In a MOR^– /–^ mouse strain, M3G hyperalgesia is abolished. **(3)** M3G can bind MD-2 and has been shown to induce the activation of the MAPK, NF-κB and AKT pathways in TLR4 signaling studies. **(4)** M3G has been described to cause the release of proinflammatory cytokines known to be powerful modulators of nociception counteracting morphine-induced antinociception. **(5)** M3G-induced hyperalgesia is also abolished in a TLR4^– /–^ mouse strain. **(6)** Interestingly, both MOR and TLR4 signaling involves the MAPK pathway. This pathway is involved in morphine-induced hyperalgesia as well as in proinflammatory cytokine release following TLR4 activation. Antagonism of the MAPK pathway components results in inhibition of M3G-induced hyperalgesia. MOR-TLR4 crosstalk might thus be involved in M3G-induced hyperalgesia. **(7)** An interesting alternative assumption suggests the existence of a yet unknown receptor that could mediated M3G effects.

The molecular mechanisms underlying the effects of M3G remain a matter of debate (Table 3). On the one hand, one study published observations in MOR^–/–^ mice suggesting its requirement for M3G pronociceptive effects (Roeckel et al., 2017). In this valuable study, i.p. administration of M3G induces thermal hyperalgesia and tactile allodynia in WT but not MOR^–/–^ animals. In addition, M3G binds MOR on brain membranes from WT mice, although with low affinity (∼1.4 μM), and induces a weak Gi-dependent activity but no β-arrestin2 recruitment (Figure 2). This activity is not observed neither in brain membranes from MOR^–/–^ mice, nor in the presence of naloxone (Roeckel et al., 2017).

**TABLE 3:** M3G pharmacological targets and effects.

References	Specie/Model	Experiment type	M3G effects
Pasternak et al., 1987	Bovine brain membranes	*In vitro*	M3G has a low affinity for MOR
Christensen and Jorgensen, 1987	Bovine brain membranes	*In vitro*	
Chen et al., 1991	Rat brain membranes	*In vitro*	
Bartlett and Smith, 1995	Sheep brain membranes	*In vitro*	
Roeckel et al., 2017	Mouse brain membranes	*In vitro*	

Labella et al., 1979	SD male rats	*In vivo*	M3G-induced hyperalgesia/allodynia is enhanced by naloxone/naltrexone treatment
Woolf, 1981	SD rats	*In vivo*	
Yaksh et al., 1986	Rats	*In vivo*	
Yaksh and Harty, 1988	Rats	*In vivo*	
Halliday et al., 1999	SD male rats	*In vivo*	

Roeckel et al., 2017	MOR^–/–^ mice	*In vivo*	MOR is required for M3G-induced hyperalgesia following i.p. injection

Lewis et al., 2010	SD male rats	*In vivo, in vitro* and *in silico*	TLR4 is required for M3G-induced hyperalgesia. M3G activates TLR4 signaling. M3G induces the release of proinflammatory cytokines.
Due et al., 2012	TLR4^–/–^ male mice and SD female rats	*In vivo* and *in vitro*	
Grace et al., 2014	SD and lewis male rats	*In vivo, in vitro* and *in silico*	
Xie et al., 2017	HEK cells	*In vitro*	
Allette et al., 2017	SD rats	*In vivo* and *in vitro*	
Doyle and Murphy, 2018	SD male and female rats	*In vivo*	
Iqbal et al., 2020	PC12 cells	*In vitro*	
Wang et al., 2021	C57BL/6 mice and human lung cancer cell lines	*In vivo* and *in vitro*	

Sullivan et al., 1989	SD male rats	*In vivo* electrophysiologi-cal recording	M3G does not affect basal or morphine-induced inhibition of C-fiber-evoked responses of convergent dorsal horn neurons, neither on membrane currents or action potential firing in locus coeruleus neurons
Hewett et al., 1993	SD male rats	*In vivo* electrophysiologi-cal recording	
Osborne et al., 2000	SD male rats	*In situ* electrophysiologi-cal recording	

Bartlett et al., 1994a	SD male rats	*In vivo*	M3G-induced behavioral excitation involves the indirect activation of NMDA receptors.
Hemstapat et al., 2003	Primary cultures of embryonic rat hippocampal neurones	*In vitro*	

Bartlett et al., 1994b	SD male rats	*In vitro*	M3G does not interact with opioid, GABA_A_, AMPA, NMDA, kaïnate or glycinergic receptors, nor alters GABA or glutamate release from synaptosomes.
Bartlett and Smith, 1996	SD male rats	*In vitro*	

Moran and Smith, 2002	SD rats	*In vitro*	M3G reduces the amplitude of GABAerbic and glycinergic inhibitory post-synaptic currents in the rat substantia gelatinosa through a presynaptic mechanism

Komatsu et al., 2009	ddY male mice	*In vivo*	i.t. M3G-induced behavioral excitation involves the ERK-NO-cGMP-PKG pathway and is blocked by coadministration of naltriben, a selective δ_2_-opioid receptor antagonist
Komatsu et al., 2016	ddY male mice	*In vivo*	

Due et al., 2014	SD male and female rats	*In vitro*	M3G-induced increase of sensory neurons excitability is blocked by carbamazepine, an inhibitor of several voltage-dependent sodium channels

Arout et al., 2014	CD-1 male mice	*In vivo*	i.p. injection of M3G induces c-Fos activation in the PAG

Juni et al., 2006	CD-1 male mice	*In vivo*	M3G induces hyperalgesia following chronic treatment with high doses but not low doses of morphine

Blomqvist et al., 2020	SD male rats	*In vivo*	Chronic i.t. injections of M3G causes antinociceptive cross-tolerance to morphine and increases substance P expression in the dorsal horn of the spinal cord

Igawa et al., 1993	SD female rats	*In vivo*	i.t. M3G injection has excitatory effects on micturition

Thomas et al., 1995	Female B6C3F1 mouse cells	*In vitro*	M3G modulates B cell proliferation

Hashiguchi et al., 1995	SD male rats	*In vivo*	M3G enhance the hyperglycemic effects of M6G

*AMPA, α-amino-3-hydroxy-5-methylisoxazole-4-propionate; CNS, central nervous system; ddY, Deutschland, Denken, and Yoken mice; DOR, δ-opioid receptor; DRG, dorsal root ganglion; ERK, extracellular signal-regulated kinase; GABA, γ–aminobutyric acid; GABA_A_, GABA receptor A; HEK, human embryonic kidney cells; KO, knock-out; KOR, κ-opioid receptor; LC, locus coeruleus; LPS, lipopolysaccharide; M3G, morphine-3-glucuronide; M6G, morphine-6-glucuronide; MD-2, myeloid differentiation factor 2; MOR, μ-opioid receptor; NF-κB, nuclear factor kappa-light-chain-enhancer of activated B cells; NMDA, N-methyl-D-aspartate; NO-cGMP-PKG, nitric oxide–cyclic guanosine monophosphate–protein kinase G signaling pathway; OIH, opioid-induced hyperalgesia; PAG, periaqueductal gray; PD-L1, programmed death-ligand 1; SD, Sprague-Dawley; TLR4, Toll-like receptor 4; vl-PAG, ventrolateral periaqueductal gray.*

On the other hand, M3G showed only low (>μM) affinity for MOR in several binding studies employing radio-labeled molecules, such as [D-Ala2, *N*-MePhe4, Gly-ol]-enkephalin (DAMGO) or naloxone (Labella et al., 1979; Christensen and Jorgensen, 1987; Pasternak et al., 1987; Coimbra-Farges et al., 1990; Chen et al., 1991; Roeckel et al., 2017). It was even proposed that the apparent affinity of M3G for MORs results from residual morphine contamination in M3G stock solutions (Bartlett and Smith, 1995). In addition, several *in vivo* studies have demonstrated that M3G’s pronociceptive effects persist in the presence of naloxone or naltrexone, two non-selective antagonists of MORs, whether they are injected systematically or directly into the CNS (Labella et al., 1979; Woolf, 1981; Yaksh et al., 1986; Yaksh and Harty, 1988; Halliday et al., 1999). Altogether, these pieces of evidence indicate that M3G might not bind to MOR, although one study suggested that this receptor appears to be mandatory for M3G effects.

#### TLR4

One interesting hypothesis suggests the existence of an alternative non-opioid receptor that could mediate M3G effects (Table 3). More precisely, *in silico* studies have indicated that M3G is able to bind the Toll-like receptor 4 (TLR4) and myeloid differentiation factor 2 (MD-2) complex through an interaction with the lipopolysaccharide (LPS) binding pocket of MD-2 (Hutchinson et al., 2010; Lewis et al., 2010; Grace et al., 2014) (for a review of opioid interactions with TLR4, see Gabr et al., 2021). TLR4 downstream signaling involves the activation of 3 parallel intracellular pathways: the NF-κB, the MAPK and the PI3K/AKT pathway. In agreement with the initial reports, it has been shown *in vitro* that the reporter cell line HEK-Blue™ hTLR4 exhibits significant activation upon M3G stimulation, which is inhibited by LPS from *Rhodobacter sphaeroides* (LPS-RS), a selective TLR4 antagonist (Lewis et al., 2010; Xie et al., 2017). This reporter cell line expresses the human TLR4 and a reporter gene under the control of a promoter inducible by NF-κB and AP-1, two transcription factors involved in TLR4 signaling cascade and proinflammatory cytokines release. In addition, it has been shown that the PI3K/AKT pathway, the third TLR4 intra-cellular signaling pathway, is also activated following M3G stimulation (Figure 2; Hutchinson et al., 2010; Wang et al., 2021). In human cancer cell lines, the activation of the AKT pathway by M3G results in upregulation of programmed death ligand 1 (PD-L1), which promotes tumor growth (Wang et al., 2021). It is, however, worth noting that, although Wang et al. (2021) observed activation of the AKT and NF-κB pathways in the A549 cell line (a human lung cancer cell line), they did not observe activation of the MAPK pathway in their model.

*In vivo*, M3G-induced hyperalgesia following i.p. administration in rodents is abolished by administration of TLR4 antagonists, as well as in a TLR4^–/–^ mouse model (Figure 2; Due et al., 2012; Allette et al., 2017). Consistently, M3G seems to display proinflammatory properties through upregulation of NF-κB and proinflammatory cytokines, including interleukin 1β (IL-1β), interleukin 6 (IL-6), and tumor necrosis factor α (TNFα), such that it was proposed to be involved in the modulation of morphine properties (Figure 2; Lewis et al., 2010; Grace et al., 2014; Doyle and Murphy, 2018; Iqbal et al., 2020; Wang et al., 2021). These interesting findings take into account that a considerable number of studies have described the immunomodulatory effects of morphine and M3G (Wybran et al., 1979; Shavit et al., 1986; Freier and Fuchs, 1994; Thomas et al., 1995; Wang et al., 2012; Eisenstein, 2019). Considered together, these data suggest that TLR4 could be responsible for the inflammation triggered by M3G, which would thwart morphine’s analgesic effects.

Several studies have implicated TLR4 in dampening morphine antinociceptive effects or in some side effects, such as antinociceptive tolerance (Hutchinson et al., 2010; Liu et al., 2011; Wang et al., 2012, 2020, 2021; Eidson and Murphy, 2013; Bai et al., 2014; Grace et al., 2014; Eidson et al., 2017; Thomas et al., 2022). For instance, a recent study revealed that antinociceptive tolerance was prevented in TLR2 and TLR4 null mutants, but not in MyD88^–/–^ animals (Thomas et al., 2022). Since several studies suggested that TLR4 could be the receptor mediating M3G effects, M3G has been proposed to play a role in morphine side effects, especially in antinociceptive tolerance (Juni et al., 2006; Blomqvist et al., 2020). However, one should note that two studies invalidate the implication of TLR4 in morphine’s effects (Fukagawa et al., 2013; Mattioli et al., 2014). The TLR4 mutant mouse strain C3H/HeJ, which expresses a non-functional TLR4, a TLR4^–/–^ mouse strain on a C57BL/6 background and the B10ScNJ mouse strain, which has a spontaneous mutation that completely removes the TLR4 coding sequence, were used. In the first study, after repeated injection of morphine, CD11b (a marker of microglial activation) mRNA expression was increased in the spinal cord of control mice. Minocycline, a microglial inhibitor, attenuated the development of morphine tolerance in these mice. Conversely, in the C3H/HeJ mutant mouse strain and in a TLR4^–/–^ mouse strain, neither the increase of CD11b mRNA expression, nor the antinociceptive tolerance was affected by TLR4 invalidation (Fukagawa et al., 2013). In the second study, neither acute antinociceptive response to a single dose of morphine, nor the development of antinociceptive tolerance was affected by TLR4 invalidation in the C3H/HeJ and B10ScNJ mouse strains (Mattioli et al., 2014). These results suggest that, in these models, TLR4 is not involved in the modulation of the antinociceptive effect of morphine, in its side effects or in the microglial activation observed during morphine tolerance. This evidence is interesting and provides insight into the complexity of M3G physiology.

#### Mu Opioid Receptor – TLR4 Crosstalk

On the one hand, M3G-induced hyperalgesia is abolished in a MOR^–/–^ mouse model (Roeckel et al., 2017). On the other hand, the same effect was observed in a TLR4^–/–^ mouse model (Due et al., 2012). This piece of evidence raises the possibility that the hyperalgesia observed following M3G administration might depend on the cross-talk between MORs and TLR4s within the CNS (Figure 2), for which both receptors are mandatory (for review, see Zhang et al., 2020). To support this idea, both receptors are expressed in microglia, astrocytes and even neurons under pathological conditions (Aicher et al., 2000; Lehnardt et al., 2003; Calvo-Rodriguez et al., 2017; Maduna et al., 2018; Zhang et al., 2018; Nam et al., 2019). Secondly, the mitogen-activated protein kinase (MAPK) pathway is recruited following both MOR and TLR4 stimulation. This pathway seems to be involved in morphine-induced hyperalgesia, as well as in the inflammatory response following TLR4 activation (Zhang et al., 2020). Finally, different studies have reported that M3G effects were abolished in presence of MAPK pathway inhibitors (Figure 2; Komatsu et al., 2009; Wang et al., 2021). Taken together, the MAPK pathway represents an interesting target to assess to better understand M3G effects.

Several studies have also suggested that, although M3G alone does not induce hyperalgesia, its coadministration with morphine decreases analgesia (Ekblom et al., 1993; Faura et al., 1996, 1997). In these studies, relatively low concentrations of M3G were injected through the i.p. route, while most of the studies in which direct hyperalgesia was observed injected high concentrations of M3G directly into the CNS (Table 1). Hence, it could be possible that, following CNS administration, M3G reaches sufficient CNS concentrations to activate both MOR and TLR4 on its own and produce hyperalgesia, although it has a low apparent affinity for MOR. In contrast, after peripheral injection of low dose of M3G alone, M3G would not reach sufficient CNS concentrations for MOR activation even though TLR4 might be activated. The presence of morphine along with M3G would then allow MOR and TLR4 activation and thus hyperalgesia. Nonetheless, this hypothesis remains to be investigated. Interestingly, in humans, M3G plasmatic and cerebrospinal fluid (CSF) concentrations following morphine administration show significant variation according to administration types, doses and patients (Hand et al., 1987b; Osborne et al., 1990; Hasselstrom and Sawe, 1993; Goucke et al., 1994; Westerling et al., 1995; Wolff et al., 1995, 1996; Hoffman et al., 1997; Christrup et al., 1999; Smith et al., 1999; Sarton et al., 2000; Meineke et al., 2002). For instance, after i.v. injection of 5 mg of morphine in healthy volunteers, M3G maximal plasmatic concentration reaches approximatively 100 nM, whereas it reaches 2 μM after a 30 min infusion of 0.5 mg/kg of morphine in neurosurgical patients (Hasselstrom and Sawe, 1993; Meineke et al., 2002). In the CSF, M3G concentrations range approximatively from 4 nM in patients that were given 30 mg of morphine orally to 0.7 μM in patients receiving chronic oral morphine therapy (Hand et al., 1987b; Goucke et al., 1994; Wolff et al., 1995, 1996; Smith et al., 1999). Depending on dose and treatment duration, M3G might reach the required CNS concentrations to induce MOR and TLR4 activation.

It is also worth noting that, although numerous studies have proposed pieces of evidence that TLR4 is involved in M3G’s effects, there is few data regarding the direct binding of M3G to TLR4. In a biophysical binding assay, M3G has been shown to bind the accessory protein MD-2 with a relatively low dissociation constant of approximatively 1.5 μM (Grace et al., 2014). However, there is no study in which radiolabeled molecules were used to investigate whether M3G can bind TLR4 or not. Therefore, one should consider an additional assumption that suggests the existence of an alternative receptor that could trigger a TLR4-dependent signaling pathway (Figure 2). In addition, to our knowledge, TLR4/MOR heteromers have not yet been described, although such association might participate in the complex response to M3G.

### Modulation of Neuronal Activity

Since the early 1990s, several studies have investigated the effects of M3G on the modulation of neuronal activity (Table 3). Consistent with the TLR4 assumption, M3G increases the excitability of nociceptive dorsal root ganglion neurons in a similar manner as LPS, and this effect seems to rely on TLR4 (Due et al., 2012, 2014; Allette et al., 2017). The implication of NaV currents has subsequently been reported in this phenomenon using carbamazepine, a known inhibitor of several NaV channels (Due et al., 2012, 2014). Concomitantly, one study showed higher c-Fos levels within the PAG following s.c. co-administration of naltrexone and M3G, rather than naltrexone and morphine (Arout et al., 2014).

Ionotropic *N*-methyl-D-aspartate (NMDA) glutamatergic receptors also appear to be involved in M3G’s effects. First, M3G did not induce any excitation when embryonic cultured hippocampal neurons were preincubated with 6-cyano-7-nitroquinoxaline-2,3-dione (CNQX, an NMDA receptor antagonist), showing the requirement of this receptor in the excitatory effects of M3G *in vitro*. This inhibition is not observed with naloxone and seems to rely on the indirect recruitment of NMDA receptors (Hemstapat et al., 2003). Moreover, behavioral excitation triggered by M3G administration was attenuated in rats pretreated with LY274614, another NMDA receptor antagonist, or when antagonists were coinjected with M3G (Bartlett et al., 1994a; Komatsu et al., 2009). Komatsu et al. (2009) have performed i.t. injections of M3G together with different antagonists, and they postulated that the phosphorylation of extracellular signal-regulated kinases (ERKs) follows the activation of the NO-cGMP-PKG pathway in response to NMDA receptor activation and that this mechanism could be responsible for an increase in neuronal excitability after M3G administration. Later, the same group showed that both nociceptive responses induced by M3G and ERK activation might be triggered via δ2-opioid receptors (DOR2) activated by Leu-enkephalin (Komatsu et al., 2016).

These data are, to a certain extent, consistent with M3G having no affinity for NMDA receptors and not being able to modulate glutamate release from whole-brain synaptosomes (Bartlett et al., 1994b; Bartlett and Smith, 1996). M3G fails to affect evoked excitatory postsynaptic currents obtained from patch-clamp recordings in neurons of the substantia gelatinosa, yet it decreases the amplitude of inhibitory postsynaptic currents in a dose-dependent manner. This effect is insensitive to naloxone and seems to stem from a presynaptic mechanism, resulting in the disinhibition of substantia gelatinosa neurons, although the identity of the recorded neurons remains unknown (Moran and Smith, 2002). This study seems to note that M3G could modulate the inhibitory systems in the spinal cord. However, it is worth noting that M3G fails to modulate γ-aminobutyric acid (GABA) release from whole-brain synaptosomes, although local suppression of GABA release, for instance, in the spinal cord, should not be excluded (Bartlett and Smith, 1996). Other reports have made this puzzling situation even more complex. Indeed, some *in vivo* pieces of evidence have suggested that M3G has no effect on the C-fiber-evoked responses of dorsal horn nociceptive neurons following i.t. pretreatment in anesthetized rats (Sullivan et al., 1989; Hewett et al., 1993; Osborne et al., 2000). Overwhelmingly, the current consensus is that M3G might modulate neuronal activity through a non-opioidergic pathway, but considerable efforts are still needed to clarify the exact underlying mechanism. Finally, M3G has also been shown to modulate several peripheral functions such as micturition and glycemia regulation following M6G administration (Igawa et al., 1993; Hashiguchi et al., 1995).

## Summary

**FIGURE 3:**
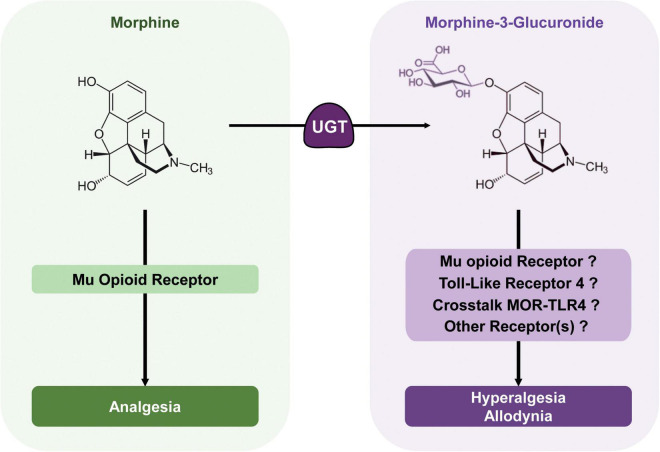
Morphine metabolic balance participates to the modulation of morphine analgesia. Morphine acts mainly on MORs to produce potent analgesia. It is metabolized by UGTs into its predominant metabolite; M3G. In rodents, M3G has been described as inducing thermal hyperalgesia and tactile allodynia, which might oppose morphine analgesia, although in humans, the relevance of these effects remains a matter of debate. M3G has been proposed to bind to TLR4, and this receptor appears to be required for M3G-induced hyperalgesia. At the same time, one study has shown that MORs are also required for M3G effects, although M3G seems to have no or little affinity for these receptors. Considered together, the crosstalk between these receptors could be key to M3G effects, whereas another receptor could also be involved.

With these outcomes considered together, M3G is able to induce both hyperalgesia and allodynia in rodents and could thus oppose morphine antinociception, although the relevance of its effects in humans is debated. M3G might act on TLR4 or both TLR4 and MOR, as well as on an additional receptor not yet characterized (Figure 3). Such a multimodal mechanism might explain the heterogeneity observed between studies and the difficulty of drawing conclusions regarding M3G neuronal effects.

## Author Contributions

FG, VH, and YG: writing – original draft. FG, VH, YG, and A-KB: writing – review and editing. YG: funding acquisition. FG and YG: supervision. All authors contributed to the article and approved the submitted version.

## Conflict of Interest

The authors declare that the research was conducted in the absence of any commercial or financial relationships that could be construed as a potential conflict of interest.

## Publisher’s Note

All claims expressed in this article are solely those of the authors and do not necessarily represent those of their affiliated organizations, or those of the publisher, the editors and the reviewers. Any product that may be evaluated in this article, or claim that may be made by its manufacturer, is not guaranteed or endorsed by the publisher.

## Abbreviations

ADME: absorption-distribution-metabolism-excretion
BBB: blood-brain barrier
CNQX: 6-cyano-7-nitroquinoxaline-2,3-dione
CNS: central nervous system
CSF: cerebrospinal fluid
DAMGO: [D-Ala2, *N*-MePhe4, Gly-ol]-enkephalin
DOR2: δ2-opioid receptors
ER: endoplasmic reticulum
ERK: extracellular signal-regulated kinases
GABA: γ-aminobutyric acid
i.c.v: intracerebroventricular
IL-1β: interleukine 1β
IL-6: interleukine 6
i.p.: intraperitoneal
i.t.: intrathechal
LPS: lipopolysaccharide
LPS-RS: LPS from *Rhodobacter sphaeroides*
M3G: morphine-3-glucuronide
M6G: morphine-6-glucuronide
MAPK: mitogen-activated protein kinase
MD-2: myeloid differentiation factor 2
MOR: μ-opioid receptor
MRP: multidrug resistance-associated protein
NF-κB: nuclear factor κB
NMDA: ionotropic *N*-methyl-D-aspartate
OATP: organic anion transporter polypeptides
OCT: organic cation transporter
PAG: periaqueductal gray
P-gp: *P*-glycoprotein
RVM: rostral ventromedial medulla
s.c.: subcutaneous
TLR4: toll-like receptor 4
TNFα: tumor necrosis factor α
UDPGA: uridine diphosphate glucuronic acid
UDP-GlcNac: UDP-*N*-acetylglucosamine
UGTrel7: UDP-galactose transporter-related protein 7
UGT: uridine 5′-diphospho-glucuronosyltransferases.
